# Advances in Antibody-Based HIV-1 Vaccines Development

**DOI:** 10.3390/vaccines8010044

**Published:** 2020-01-25

**Authors:** Ursula Dietrich

**Affiliations:** Georg-Speyer-Haus, Institute of Tumor Biology and Experimental Therapy, Paul-Ehrlich-Str. 42-44, 60596 Frankfurt, Germany; ursula.dietrich@gsh.uni-frankfurt.de

Despite the great success of antiretroviral therapy, both in the treatment and prevention of HIV-1 infection, a vaccine is still urgently needed to end the epidemic. According to UNAIDS, in 2018, about 35% of HIV-1 infected persons did not receive antiretroviral therapy (ART), resulting in 1.7 million new infections in that year. One major reason for actual HIV-1 transmissions is the fact that about 20% of HIV-1 infected persons do not know about their HIV-positive status and therefore are not on ART despite the potential high viral load linked to high risk of virus transmission. Therefore, in particular in countries with high HIV incidence, a preventive vaccine is required to reduce HIV transmissions in the general population. In this special issue of *Vaccines,* invited experts contribute a series of articles to the current understanding of antibody-based HIV-1 vaccine development.

HIV vaccine development has a long history of negative results, which nevertheless helped to accumulate detailed insights into the underlying reasons for their failure. Due to the tremendous intrinsic variability of HIV-1, the epidemic is dominated by multiple subtypes and recombinant forms, which circulate worldwide. Within an infected person, viral variants continously evolve from the transmitted/founder virus into a divergent “quasispecies” allowing their escape from the antiviral pressure imposed by the immune response or antiretroviral therapy (ART). In the paper by Stefic et al., the authors stress the importance of considering transmitted/founder viruses in vaccine development and in neutralization evaluation studies, as these are primarily the viruses a preventive vaccine has to target [1]. Interestingly, the coevolution of the transmitted/founder virus with the mounted immune response gradually leads to escape mutants with an increased antibody resistance over time, not only within patients, but also at the population level.

Intensive collaborative work in the last decade resulted in the isolation of antibodies from a subset of HIV-positive patients, that are able to potently neutralize a broad spectrum of primary (i.e., patient-derived) HIV-1 isolates in vitro and to protect from infection in animal models, underlining the importance of such broadly neutralizing antibodies (bnAbs) as correlates of protection. However, despite the fact that many different HIV-1 Env immunogens have been studied in non-human primates and in clinical trials, no bnAbs could be induced so far upon vaccination. Nevertheless, recent advances in single B cell cloning in conjunction with next generation sequencing have allowed the study of the gradual development of bnAbs in patients over time. The review by Kreer et al. from the group of Florian Klein, one of the pioneers of this work, excellently summarizes how B cell receptor variability is generated and, most importantly, how B cell receptor analysis in patients developing bnAbs can guide vaccination strategies to induce such antibodies [2]. It is crucial to identify Env immunogens targeting the germline B cell receptors, but also to identify intermediate Env immunogens, which allow to sequentially boost the initial response towards the development of bnAb characterized by special features like long HCDR3 loops, hypermutations, insertions and deletions and sometimes unconventional modes of antigen binding.

Although this sophisticated type of bnAbs against HIV-1 has not been induced thus far upon vaccination in humans or non-human primates, they could be induced in camelids. Camelids, besides conventional antibodies, encode heavy-chain-only antibodies, the variabel domain of which is called VHH or nanobody. Weiss and Verrips summarize here the promising work on the selection and characterization of broadly neutralizing nanobodies against HIV-1 that has been performed in recent years [3]. Due to their small size (1/10 of conventional antibodies) and their particular features, resembling those of bnAbs (long HCDR3), nanobodies can penetrate into clefts like the CD4 binding side in Env, thereby potently neutralizing HIV-1. Their small size also allows the conjugation of several nanobodies into one molecule, linkage to Fc-mediated effector functions or easy expression from various vectors suited for immune prophylaxis.

Numerous preclinical vaccination approaches have been performed with the aim to induce bnAbs against HIV-1 using various soluble or vector-expressed Env antigens. However, so far, at best, neutralizing antibodies were induced against easy to neutralize HIV-1 strains (Tier 1, mostly lab-adapted) or against autologous HIV-1 strains. One reason for the lack of induction of Abs with broad neutralizing activity against more difficult to neutralize patient-derived HIV-1 strains (Tiers 2 and 3) is certainly due to the complex in vivo maturation pathways of bnAbs described above, which are generated by the evolving HIV-1 quasispecies in patients. Nevertheless, promising vaccine vectors have been developed in these studies, and their pros and cons have been studied. The contribution of Wilmschen et al. in this issue nicely elaborates on the different vector systems and their optimization to achieve long-lasting antiviral immune responses, while minimizing antivector immunity and side effects [4]. Once suited serial Env immunogens have been identified, which are able to engage germline precursor B cell receptors as well as critical intermediate B cell receptors on the maturation pathway of bnAbs in vivo, viral vectors expressing those immunogens are available to try to trigger the same maturation pathways upon vaccination.

Besides viral vectors, nanoparticles have been shown to be suitable carriers of viral immunogens for the induction of protective antibodies, i.e., against the human Hepatitis B Virus (HBV) and the human Papilloma Virus (HPV). Likewise, the highly repetitive expression of HIV-1 Env antigens on nanoparticles allows strong B cell activation via BCR crosslinking in the B cell follicles, which is particularly important for the very rare B cell precursors of bnAbs. Brinkkemper and Sliepen excellently summarize the different studies of nanoparticles as carriers of HIV-1 Env immunogens in vaccination studies, some of which are very promising and have advanced to phase 1 clinical trials [5].

Advances in reverse vaccinology based on bioinformatic tools and the phage display technology have also led to the identification of short peptides mimicking linear or conformational epitopes as targets of bnAbs against HIV-1. As such, these peptides represent the minimal antigenic Env components relevant for vaccine development, which, however, have to be coupled to carriers to increase their antigenicity. Combadière et al. report on the actual status of HIV-1 peptide vaccines, most of which are derived from the transmembrane protein gp41 [6]. One of these peptide vaccines has already advanced to phase 2 clinical trials. Besides Env, peptides from the transactivator of transcription protein Tat have been analyzed in preventive and therapeutic vaccination studies after the observation that anti-Tat antibodies protect from disease progression. Preclinical vaccination studies in monkeys and clinical trials are comprehensively reviewed by Cafaro et al. in this issue, and support the importance of anti-Tat antibodies not only in reducing viremia, but also in impairing immune functions that promote viral replication, like immune activation and reservoir formation [7].

So far, the only clinical trial in the HIV-1 vaccine field showing a moderate reduction in HIV-1 acquisition was the RV144 prime-boost study performed in Thailand about 10 years ago. The detailed analysis of non-infected and infected individuals from this trial in the following years identified antibodies against the second variable region in Env (V2) to correlate with protection in conjunction with low IgA/IgG serum levels. The paper by Duerr and Gorny nicely summarizes the complexity of V2-specific antibodies mounted during natural infection or after vaccination [8]. Four different classes of antibodies against V2 can be induced, which differ functionally with respect to their capacity to neutralize the virus or to mediate antibody effector functions, which connect the adaptive to the innate immune system. Nevertheless, we still have to await the results of ongoing clinical trials to decipher the importance and the mechanisms of protection of vaccine-induced, V2-specific antibodies.

Interestingly, the RV144 vaccine trial mentioned above identified Fc-mediated effector functions of antibodies that correlate with protection. By attracting components of the innate immune system, such Fc-mediated effector functions can also target infected cells, thus contributing to a reduction in viremia. Anand and Finzi further explore in their excellent review the viral and cellular mechanisms affecting the different Fc-mediated effector functions, and their contribution to the control of viremia [9]. In this context, the article by Ruprecht et al. underlines the importance of mucosal effector mechanisms mediated by different IgG subclasses in protecting from mucosal infection in passive immunization studies using the simian-human immunodeficiency virus (SHIV) macaque model [10]. Whereas the IgGA1 subclass of a neutralizing antibody targeting a V3 epitope in Env protected 83% from mucosal infections, the IgGA2 subclass with the same paratope only protected 17% of the animals. Authors postulate that particular structural features of IgA1 antibodies lead to large virus–antibody aggregates resulting in immune exclusion, thereby preventing infection at mucosal barriers.

Altogether, the articles in this issue highlight different, important aspects of antibody-based HIV-1 vaccine development. Besides antibody-mediated neutralization, which is usually the protective mechanism against viral infections, antibody-mediated effector functions also play an essential role in controlling HIV-1 infection. Although the individual components necessary to develop an HIV-1 vaccine, like bnAbs, structural knowledge of their epitopes, knowledge of the antibody effector functions and a series of vaccine vectors, are known, bnAbs against primary HIV-1 strains have not yet been induced by the current Env immunogens. However, increasing knowledge on the complex maturation pathways of bnAbs in infected persons in recent years has set the basis to derive a series of recombinant Env immunogens able to engage the germline B-cell receptors of bnAbs and subsequent B cell receptors on the affinity maturation pathway towards the generation of bnAbs. Thus, learning from nature in conjunction with the development of suited techniques has rapidly advanced antibody-based HIV-1 vaccine development in the last years, providing hope that we might be a big step closer to an HIV-1 vaccine.
